# Non-invasive Neuromodulation of Arrhythmias

**DOI:** 10.19102/icrm.2024.15022

**Published:** 2024-02-15

**Authors:** Johnathon Rast, Daniel Sohinki, Alexander Warner

**Affiliations:** 1 Medical College of Georgia, Augusta, GA, USA

**Keywords:** Arrhythmias, atrial fibrillation, neuromodulation, ventricular tachycardia

## Abstract

Dysfunction of the cardiac autonomic nervous system (CANS) is associated with various cardiac arrhythmias. Subsequently, invasive techniques have successfully targeted the CANS for the treatment of certain arrhythmias, such as sympathetic denervation for ventricular tachycardia storm. Non-invasive strategies capable of modulating the CANS for arrhythmia treatment have begun to gain interest due to their low-risk profile and applicability as an adjuvant therapy. This review provides an evidence-based overview of the currently studied technologies capable of non-invasively modulating CANS for the suppression of atrial fibrillation and ventricular arrhythmias.

## Introduction

The cardiovascular and autonomic nervous systems share a complex network of structures and pathways, collectively termed the cardiac autonomic nervous system (CANS). Derangements in CANS function are known to promote the initiation and maintenance of many cardiac arrhythmias.^1^ As a result, procedures capable of invasively targeting the CANS have demonstrated success in treating various arrhythmias, such as atrial fibrillation (AF) and ventricular arrhythmias (VAs). Despite the success of these procedures, they carry risks of operative complications and therapeutic failure. Evidence is emerging around novel non-invasive strategies to modulate the CANS for the treatment of these arrhythmias. In this review, we will summarize the current evidence and propose objectives for future research regarding non-invasive modulation of the CANS for arrhythmia management.

## Cardiac autonomic nervous system overview

**Figure 1:**
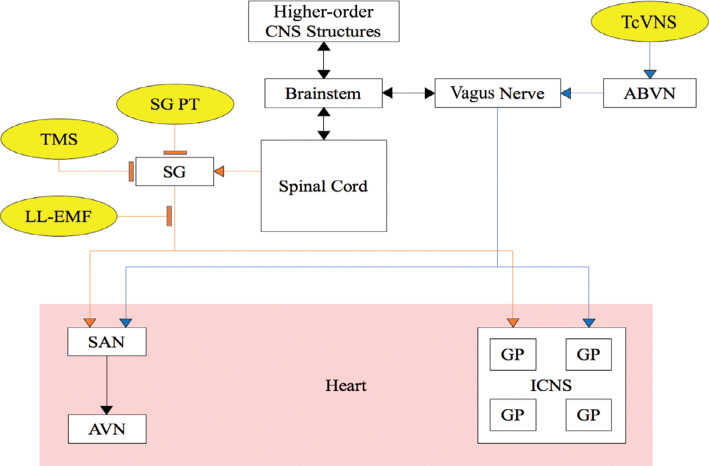
Targets for non-invasive neuromodulation of the cardiac autonomic nervous system. A broad overview of the cardiac autonomic nervous system pathways with targeted locations and actions of therapeutic devices. Lines ending in arrows and flat boxes represent activation and inhibition functions, respectively. Blue lines involve parasympathetic pathways. Orange lines involve sympathetic pathways. Black lines depict pathways involving sympathetic and parasympathetic pathways. *Abbreviations:* ABVN, auricular branch of the vagus nerve; AVN, atrioventricular node; CANS, cardiac autonomic nervous system; CNS, central nervous system; GP, ganglionated plexi; ICNS, intrinsic cardiac nervous system; LL-EMF, low-level electromagnetic field; PT, phototherapy; SAN, sinoatrial node; SG, stellate ganglion; TcVNS, transcutaneous vagal nerve stimulation; TMS, transcutaneous magnetic stimulation.

The CANS can be divided into the following three major anatomical regions: (1) extrathoracic, including structures in the central nervous system and peripheral neurons; (2) intrathoracic extracardiac; and (3) intrinsic cardiac—namely, the ganglionated plexi (GPs) on the epicardial surface and the neural pathways that permit their crosstalk.^2,3^ Each level of the CANS contains both sympathetic and parasympathetic components.^4,5^ Various brainstem nuclei house the preganglionic sympathetic neurons, whose axons travel caudally to synapse on the postganglionic sympathetic neurons adjacent to the cervical and thoracic spinal cord segments.^3,6^ One set of these ganglia, the stellate (cervicothoracic) ganglia, is considered the gateway to sympathetic response in the CANS, and thus has been targeted for various neuromodulating strategies, as depicted in **Figure 1**.^3,6–11^ Axons from these ganglia travel toward the heart and synapse on the myocardium, sinoatrial node (SAN), atrioventricular node (AVN), and adrenergic cardiac neurons.^3,6,12^ Sympathetic and parasympathetic cardiac afferent signals originate from the heart itself, the great vessels, and the coronaries.^12–14^ They are known to promote pro-inflammatory responses and assist in vascular tone regulation.^13,14^

The parasympathetic branch of the CANS also starts in the brainstem, where preganglionic neurons are housed. From there, they travel caudally via the vagus nerve, vagosympthetic trunks, and intrathoracic nerves before synapsing on the GPs located within epicardial fat pads.^3,6,12^ Signals are integrated here within the intrinsic cardiac nervous system (ICNS) before finally innervating the SAN, AVN, and myocardium.^3,6,12,15^ Afferent parasympathetic CANS pathways are initiated from vascular chemoreceptors, baroreceptors, or within the ICNS.^3,6^ These signals are then relayed through specific parasympathetic ganglia (depending on the anatomic origin of the afferent signal) before eventually synapsing at the nuclei in the medulla.^3,6^

Additionally, the GPs within the ICNS coordinate a heavily interconnected neural network.^2,6,15–17^ Signals between the heart itself and the CANS are largely regulated by these GPs and their interconnecting pathways.^2,3,6,16,18^ The GPs exhibit synaptic plasticity, giving them powerful capabilities to regulate ICNS signaling.^19^ Therapeutic techniques that induce neuromodulation of the GPs produce lasting results once the therapy ends due to a memory function within the GPs.^2,20^ All major levels of the CANS also possess multiple regulatory feedback loops via received and integrated signals from other CANS structures.^3,14,16,17^

## The cardiac autonomic nervous system and arrhythmias

Dysfunction of the CANS is associated with multiple cardiac arrhythmias, and cardiovascular disease leads to maladaptive CANS remodeling at all of its major divisions.^1,5^ The GPs mediate the dissemination of CANS dysfunction due to their interactions with afferent, efferent, sympathetic, and parasympathetic signaling.^18^ This review will focus on AF and VAs due to their predominance in the literature of the emerging field of non-invasive neuromodulation.

AF requires a trigger for induction as well as a substrate for maintenance, with both sympathetic and parasympathetic dysfunction contributing to these processes.^1,21–24^ The classic “AF begets AF” paradigm holds true, with autonomic dysfunction and remodeling promoting induction of AF, and AF subsequently promoting further sympathetic hyperactivity and CANS dysfunction.^25,26^ Excess sympathetic activation induces atrial tissue remodeling, fibrosis, and atrial electrophysiologic changes, such as increased atrial propensity to triggered activity via both early and delayed afterdepolarizations.^6,21,25–28^ These changes facilitate onset, progression, and maintenance of AF.^21,25,26,28^ A hyperactive CANS also increases norepinephrine, epinephrine, and acetylcholine release onto atrial GPs, increasing GP input into the epicardium.^7,29,30^ Due to the immediate proximity of several GPs to the pulmonary venous (PV) ostia, this promotes PV firing (as well as likely non-PV trigger induction) and initiation of AF.^26,29–31^ Chronic sympathetic predominance also leads to chronic systemic inflammation, a known risk factor for AF progression, at least partially due to the promotion of atrial fibrosis, abnormal glycogen deposition and metabolism, and fatty infiltration of the atrial wall.^28,32–35^ Additionally, sympathetic predominance leads to a reduction in parasympathetic tone, permitting a relatively unopposed pro-inflammatory state.^26,34,36^

Strong vagal responses are also capable of inducing AF.^37^ Strong parasympathetic activation decreases the atrial effective refractory period (ERP) and action potential duration.^6^ This effect occurs heterogeneously throughout the atrium, especially in the case of a scarred and remodeled atrial myocardium, leading to an increased dispersion of refractoriness.^6^ In turn, this increases the likelihood of a triggered impulse encountering unidirectional conduction block and conduction slowing, subsequently resulting in functional re-entry, which is the hallmark of AF.^6,28,30^ As such, a sudden sympathovagal response is capable of inducing AF.^23,24^

CANS dysfunction is also associated with VAs. Sympathetic hyperactivity is closely involved in the onset and maintenance of VAs, especially ventricular tachycardia (VT) and ventricular fibrillation.^38–41^ The pathophysiology shortly after an acute myocardial infarction (MI) involves excessive sympathetic activation, frequently leading to VAs.^1,40,41^ After an MI or VT episode, nerve sprouting, sympathetic hyperinnervation, and other kinds of maladaptive autonomic and myocardial remodeling frequently occur, promoting ventricular electrical instability and more VAs.^39–43^ This excessive sympathetic activity results in a shorter ventricular ERP and a greater magnitude of heterogeneity in ventricular depolarization.^6,11^ The post-MI state is associated with profound inflammation, likely also driven by a sympathetic surge, as sympathetic tone intensity appears to correlate with the magnitude of the inflammatory response.^6,44^

Multiple modalities have emerged in an attempt to modulate the CANS in order to suppress arrhythmias. Vagal nerve stimulation (VNS) is one such modality. For several decades, applying VNS at relatively high voltages was used to induce AF in experimental models.^6,7,37^ Then, in 2009, Li et al. discovered that low-level vagal nerve stimulation (LL-VNS), when performed at intensity levels below the threshold measured to begin reducing the heart rate (HR), was capable of suppressing AF onset and duration in dogs.^45^ This seemingly paradoxical phenomenon was supported by subsequent animal and human studies.^28,31,46–48^ LL-VNS triggers an efferent vagal response down the vagus nerve, terminating at the atrial GPs and suppressing their activity.^26,28,31,45,46^ This results in multiple anti-arrhythmic and antisympathetic mechanisms, including increases in atrial ERP, AF cycle length, AF induction threshold, heart rate variability (HRV), and beneficial atrial remodeling.^28,31,47,49^ This GP suppression also results in decreases in AF-inducing ectopic firing from the GPs, total time spent in AF, average length of AF paroxysms, low-frequency/high-frequency (LF/HF) ratio in HRV spectral analysis, serum tumor necrosis factor (TNF)-α level, serum C-reactive protein (CRP) level, stellate ganglion (SG) activity, and maladaptive atrial remodeling.^26,28,29,31,42,47–49^ The effects from LL-VNS include both modest parasympathetic activation and sympathetic suppression as well as the orchestration of changes in CANS pathways, creating lasting, memory-like alterations.^2,20^

## Transcutaneous vagal nerve stimulation

### Background and overview

**Figure 2:**
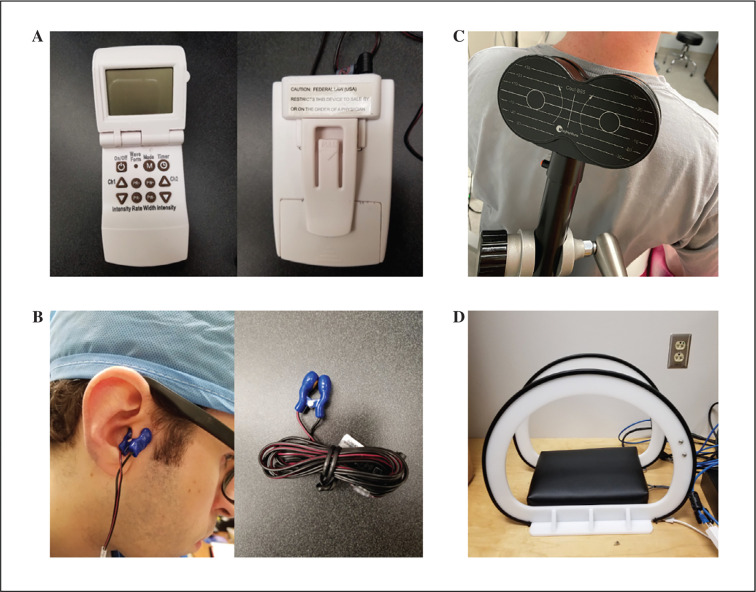
Images of transcutaneous vagal nerve stimulation (TcVNS), transcutaneous magnetic stimulation (TMS), and low-level electromagnetic field (LL-EMF) devices. The non-invasive neuromodulation devices, including the TcVNS handheld device (A), TcVNS tragus clip and wire (B), TMS device (C), and LL-EMF device (D), are illustrated. Photos of the stellate ganglia phototherapy device can be found in the original investigation published by Nonoguchi et al.^10^

The many benefits of VNS already described were previously only available through surgically implanted devices. This method carries significant operative risk and has been largely reserved for research in animal models, having been applied only sparingly in humans.^37^ Eventually, Yu et al. demonstrated a revolutionary, non-invasive modality for vagal stimulation, known as transcutaneous vagal nerve stimulation (TcVNS), as depicted in **Figure 2**.^50^ Therapy performed with this device retained the benefits of invasive LL-VNS but without the operative risks in their animal model.^50^ Application of this non-invasive VNS approach was quickly translated into human studies with similar beneficial results, showing promise as a more usable, affordable, and preferential modality due to reductions in both risk and cost to the patient.^28,48,49^ TcVNS activates the auricular branch of the vagus nerve (ABVN) through transcutaneous electrical stimulation at the tragus.^28,48,49,51–53^ The ABVN predominantly activates an afferent vagal pathway, but the eventual efferent response appears to be equivalent to invasive LL-VNS, which targets afferent and efferent vagal pathways.^28,44,48,51^ The ABVN directly transmits to the nucleus tractus solitarius in the medulla via afferent vagal pathways to the brainstem.^51,53^ Signals may then relay through various neural pathways,^53^ including an efferent tract down the vagus nerve, producing equivalent parasympathetic, anti-arrhythmic results as compared to invasive LL-VNS.^26,28,31,44–46,48,49,51,52^

### Atrial fibrillation

The first sham-controlled randomized controlled trial (RCT) using TcVNS to suppress AF in humans was published by Stavrakis et al., who demonstrated that AF inducibility and duration were suppressible after just 1 h of treatment with TcVNS, as illustrated in **Table 1**.^28^ They also measured an increase in AF cycle length, an increase in the threshold for AF induction, a decrease in atrial ectopy, and an increase in the atrial ERP in the right atrium and coronary sinus, all of which represent electrophysiologic changes that suppress AF.^28^ Additionally, these authors observed a significant reduction in systemic serum TNF-α and CRP levels.^28^ These measurable improvements after just one treatment interval imply a powerful capacity for TcVNS to modulate the CANS. Stavrakis et al. followed up on these positive results with completion of the Transcutaneous Electrical Vagus Nerve Stimulation to Suppress Atrial Fibrillation (TREAT AF) trial, a longer-term sham-controlled RCT studying the intermittent daily use of TcVNS in an ambulatory setting.^48^ Here, they demonstrated that daily intermittent use of ambulatory TcVNS over a 6-month period reduced the total time spent in AF, reduced atrial ectopic beats, increased the LF/HF ratio in HRV spectral analysis, and reduced serum TNF-α levels.^48^ However, they did not observe a significant reduction in the longest paroxysm of AF averaged across each group.^48^ These investigators additionally suggested that TcVNS suppresses the inducibility of AF, but it may not suppress the maintenance of an AF paroxysm.^48^ However, they gathered conflicting data in the first human RCT using TcVNS to suppress AF, which suggested that TcVNS alters atrial electrophysiology in ways that facilitate the early termination of AF.^28^ The reliability of the TREAT AF trial findings is in part limited by a small sample size of 53 patients. Larger human randomized trials may clarify whether TcVNS can suppress the maintenance of AF.

### Ventricular arrhythmias

Beyond AF, TcVNS also appears to suppress VAs, likely as a consequence of suppressing sympathetic tone, reducing systemic inflammation, and mitigating proarrhythmic remodeling of the GPs and myocardium that occurs following acute MI.^42,44,49^ Despite the immense benefit of percutaneous coronary intervention, the resulting reperfusion injury remains a therapeutic challenge as it can promote further myocardial injury and VAs. TcVNS reduces sympathetic activation, suppressing ischemia-related and reperfusion-related VAs shortly after acute MI. In animal models, invasive LL-VNS was first discovered to suppress VAs after acute MI.^54^ Subsequent animal studies also showed VA suppression with the non-invasive TcVNS.^42,43^ More specifically, Yu et al. found that applying TcVNS for 2 h daily for 2 months in dogs immediately following acute MI suppressed reperfusion-related VAs, maladaptive autonomic remodeling at the border zone of infarcts, and sympathetic tone via reduction of SG activity.^42^

Subsequently, Yu et al. randomized 95 human patients who presented with acute ST-segment–elevation MI within 12 h of symptom onset to receive TcVNS or a sham control for 2 h immediately after reperfusion.^44^ The experimental group experienced a significant reduction in episodes of VT and premature ventricular complexes.^44^ However, VT episodes were not further subclassified as sustained or non-sustained, clouding the certainty of clinical improvement. Patients in the experimental arm also saw a significant reduction in serum creatinine kinase–myoglobin binding and the levels of myoglobin, N-terminal pro-B-type natriuretic peptide, TNF-α, interleukin-1β, and interleukin-6 as well as an increase in both the left ventricular ejection fraction (LVEF) and wall motion index.^44^ Collectively, these findings suggest that TcVNS may provide a significant clinical benefit. However, larger randomized studies that measure episodes of sustained VT as well as mortality rates are needed to confirm a significant benefit from TcVNS use for VT-predisposed patients.

### Benefits and uncertainties

Each non-invasive technique discussed in this review, including TcVNS, did not require alterations in standard anti-arrhythmic therapy. Thus, they can be added to standard therapy instead of replacing another component. The advantage of rapid, lasting therapeutic benefit is thought to be a consequence of the synaptic plasticity of the interconnected GP, which provides a capacity for “rewiring” and a “memory” effect.^19,28,48^ Although data suggest that repeated TcVNS use carries a clinical benefit, the temporal therapeutic window for optimal results will need clarification in future research.

The target therapeutic TcVNS voltage must be measured to begin treatment, as it differs between individuals. It can be quickly found through non-invasive voltage titration. The two methods used for voltage titration are conduction slowing and patient discomfort thresholds. Voltages selected for therapy that were successful were set to 50%–80% of the measured threshold voltage.^28,44,48,50^ The voltage threshold that begins causing patient discomfort appears to be similar, if not slightly greater, than the threshold of SAN or AVN slowing.^28^ Even if the cardiac conduction slowing threshold is lower than a patient’s discomfort threshold, titrating the voltage upward to discover the discomfort threshold is likely still safe in most patients.^28,44,48^ VNS appears to only induce AF at voltage intensities capable of slowing the HR by 40%.^55^ After identifying an appropriate device voltage for patients, TcVNS can then be used in the ambulatory setting.

The data from TREAT AF strongly suggest that there may be TcVNS responders and non-responders.^48^ Thus, a biomarker that correlates with treatment efficacy would be greatly beneficial for patient selection.^48^ In an ancillary study of TREAT AF, Kulkarni et al. found that P-wave alternans (PWA), an electrocardiographic phenomenon associated with AF risk, may serve this function.^52^ Kulkarni et al. found that the acute response to TcVNS caused opposing effects on PWA based on whether patients were receiving chronic TcVNS or not.^52^ The TREAT AF experimental arm saw a reduction in PWA from acute and chronic TcVNS.^52^ However, the sham-control arm, which received only acute TcVNS therapy, resulted in a transient increase in PWA.^52^ Larger studies of chronic and acute TcVNS use while measuring PWA, and therapeutic response will be required to confirm the utility of PWA as a biomarker for TcVNS efficacy. Multiple other biomarkers have been proposed to evaluate responsiveness to TcVNS, including HRV, global longitudinal strain, serum TNF-α, serum CRP, and serum neuropeptide Y.^4,7,28,32,36,48,56^ The correlative strength between each potential biomarker and TcVNS treatment efficacy must be further explored before any of them can guide clinical management.

Regarding patient populations, paroxysmal AF is the only AF subtype that TcVNS has been studied on thus far, though CANS dysfunction is implicated in persistent AF as well.^25^ Thus, TcVNS may be able to reverse the electrophysiologic remodeling that sustains AF in persistent patients, though this will require studies to confirm. Additionally, nearly all human RCTs using TcVNS for arrhythmias exclude patients suffering from heart failure with reduced ejection fraction (HFrEF) and LVEFs of <40%.^28,48,52^ The one exception still excluded patients with severe systolic heart failure, defined by LVEF < 30%.^44^ These patients were likely excluded to eliminate a potential confounding variable, as there are conflicting data regarding the benefit of TcVNS for HFrEF. Eventually, future studies should consider evaluating the benefits of TcVNS in patients with HFrEF and either AF or VAs, as they may receive an additional benefit.

## Low-level electromagnetic field application

As has been noted previously, non-invasive stimulation of the vagus nerve shows promise as a therapeutic modality in the treatment of AF. One novel approach involves the application of a low-level electromagnetic field (LL-EMF) to the patient’s body to affect autonomic stimulation. The rationale for this approach has its origins in special relativity, which posits that, for a given molecular target, there exists an LL-EMF field strength and frequency which, if applied to a biological system, can stimulate that molecular target.^57^ The derivation of the required calculations is beyond the scope of this review, but its application allows the user to tailor the device settings to amplify biomolecules of interest, such as proteins related to inflammation.^57^

Multiple animal models have demonstrated a salutary effect of LL-EMF in treating neuropathy and wound healing, showing improvements in strength, axonal myelination, and wound apposition.^57–59^ In these studies, LL-EMF strength and frequency were calculated to target effector molecules such as nerve growth factor, dynein, kinesin, and acetylcholine. These studies provide a proof of concept for the biological impact of LL-EMF and raise the possibility of using LL-EMF as a therapy for cardiovascular disease.

Preliminary data do suggest an effect of LL-EMF on the cardiovascular system. Scherlag et al. were able to demonstrate the slowing of AVN conduction as well as both the induction and suppression of atrial arrhythmias in response to LL-EMF delivered with various parameters in a dog model.^60^ Further, these effects were attenuated by the administration of pharmacologic autonomic blockade, lending further evidence to an autonomic effect of LL-EMF. While field strength and frequency were empirically derived in these initial studies, Yu et al. were able to use specifically calculated parameters to suppress pacing-induced AF in a dog model.^61^ The molecular weight of canine vasostatin-1 was used to calculate appropriate field parameters, with a reduction in pacing-induced AF noted as well as changes in atrial refractoriness noted in response to targeted LL-EMF stimulation.^61^

These data have since been translated into humans. Microtesla-level LL-EMF has been demonstrated to affect reductions in overall HR as well as increases in both time and frequency domain measures of HRV, correlating with decreases in sympathetic and increases in parasympathetic tone.^62–65^ These preliminary data have served as a proof of concept for studies using LL-EMF in the treatment of cardiovascular disease. Sohinki et al. recently investigated the ability of LL-EMF to attenuate pacing-induced AF in patients presenting for AF ablation.^66^ In response to 60 min of LL-EMF stimulation applied over the head and neck, they were able to demonstrate reductions in the duration of pacing-induced AF (11.0 ± 3.43 min; *P* = .03), ectopic firing initiating spontaneous episodes of AF, and reductions in levels of the chemoattractant molecule monocyte chemoattractant protein-1.^66^

LL-EMF has several obvious advantages and disadvantages as a therapeutic modality. No adverse effects of LL-EMF at the previously studied field strengths have ever been reported, making it an attractive option for patients who are at high risk or who have preferences against medical or invasive therapy. However, options for delivering therapy are currently cumbersome, limiting their application in both the inpatient and outpatient settings. While the initial data reported are promising, further studies examining clinical outcomes in patients with both atrial arrhythmia and VA are required before LL-EMF becomes a mainstream therapy for tachyarrhythmias.

## Transcutaneous magnetic stimulation

Another non-invasive electromagnetic field-induction device, which uses a magnetized figure-of-eight coil placed slightly above the skin adjacent to the targeted neural structure, has demonstrated therapeutic utility in multiple diseases, including epilepsy, multiple sclerosis, depression, anxiety, and chronic pain.^67^ This therapy, termed transcutaneous magnetic stimulation (TMS), has also recently demonstrated a therapeutic benefit for suppressing VAs when positioned to target the left SG.^8,9^ As has been previously noted, hyperactivity of the left SG can be arrhythmogenic in at-risk patient populations. TMS modulates the CANS by suppressing the left SG activity, resulting in sympathetic tone reduction and VA suppression. Knowing this role of autonomic dysfunction in VAs, Wang et al. studied the effect of TMS targeting the left SG on VAs in a post-MI population of dogs.^68^ They found a significant reduction in sympathetic tone, left SG activity, and VAs.^68^ Markman et al. first studied TMS in humans with VAs via a five-patient case series assessing the impact of TMS in patients hospitalized with VT storm.^8^ Patients received TMS over the left SG once for 1 h, and various VA parameters recorded during the 24 h before treatment and throughout the 72 h after treatment were analyzed.^8^ Despite the small population size, their results were striking. All patients had several episodes of sustained VT before treatment, averaging 19.8 episodes per patient in the 24 h before receiving TMS.^8^ Twenty-four hours after treatment, only one patient had any sustained VT episodes (5 episodes); it is worth noting that this patient had 53 episodes of sustained VT during the 24 h before treatment.^8^ During the 25–28 h post-treatment, all patients remained free of sustained VT episodes.^8^ At final follow-up (49–72 h), four of five patients remained free of sustained VT.^8^

To follow up on the positive results from this case series, this same research group published a sham-controlled RCT evaluating the efficacy of TMS in patients hospitalized for VT storm.^9^ Although this study did not meet its primary endpoint of freedom from sustained VT over the first 24 h following treatment, they did meet a secondary endpoint of significantly reduced sustained VT episodes over the first 72 h following TMS treatment.^9^ Of note, unlike in the study performed by Yu et al.,^44^ the VT data-collection model used by Markman et al. separated non-sustained VT from sustained VT.^8,9^ This allowed for a better assessment of the correlation between VA reduction and patient prognosis. Several directions for TMS research may yield more positive results moving forward. For example, studying VA suppression in acute MI patients with TMS is a promising next step in research progression. Another population of interest is patients with HFrEF and LVEFs of <35% due to their relatively high risk of VAs and autonomic dysfunction. Additionally, intermittent use of TMS for the primary prevention of VAs could perhaps reveal a supplemental role for TMS in arrhythmia management in at-risk patient populations.

Similar to TcVNS, one uncertainty that should be prioritized in future studies is optimal device settings and parameters. If there is no specific intensity of magnetism that optimizes therapy, clinicians should be informed of an intensity range that constitutes a known therapeutic benefit. Additionally, TMS is typically used in intervals, not just once, in other diseases that benefit from the therapy. Future studies should attempt to uncover the optimal quantity, density, and length of therapeutic sessions with TMS. Defining optimal parameters will strengthen the reliability of any studies that reveal no benefit, and it will more precisely reveal the magnitude of benefit in diseases studied that positively respond to TMS.

## Stellate ganglion phototherapy

The left SG, the gateway to the sympathetic CANS, has become a target for inhibition in conditions propagated by sympathetic overdrive. Bilateral sympathetic denervation, an invasive procedural ablation of left SG innervation to the heart, can provide benefit for patients suffering from VT storm.^38^ While this procedure is considered minimally invasive because of its endovascular approach, it still carries a perioperative risk.^38^ Alternatively, non-invasive blockade of the left SG can also be performed via irradiation with low-level lasers, a technique termed SG phototherapy. SG phototherapy can improve various conditions characterized by autonomic dysfunction with sympathetic predominance, including chronic pain syndromes, tinnitus, and hyperhidrosis.^69^

As phototherapy is capable of blocking SG activity and other methods of SG blockade can provide benefit in VT storm, Nonoguchi et al. investigated whether SG phototherapy could suppress VAs in patients with refractory VT storm.^10^ They performed a non-randomized, two-part cohort study to test their hypothesis with healthy volunteers and then patients with refractory VT storm.^10^ Each participant underwent 10 min of bilateral SG phototherapy for a total of eight sessions over a 1-month period.^10^ The two devices used in the protocol collectively emitted light wavelengths of 400–1600 nm, falling inside the visible light and infrared spectra.^10^ The healthy subjects demonstrated evidence of reduced sympathetic tone and had decreases in serum catecholamines, but none of these changes were sustained at follow-up 3 months after the protocol first began, suggesting that the changes from SG phototherapy may not cause enduring neuromodulation.^10^ SG phototherapy nearly reached a significant reduction in VA burden in this study of 11 patients in VT storm—it reduced the VA burden from 8.0 ± 9.0 episodes per day to 2.0 ± 5.3 episodes per day (*P* = .066).^10^ Nonoguchi et al. suggested that SG phototherapy benefits may be strongest in the acute timeline and may serve as a bridge to sympathetic denervation or catheter ablation rather than as a replacement therapy.^10^ Although SG phototherapy is generally safe and may offer an acute benefit for patients in VT storm, any clinical benefit must be verified with larger studies.

Another promising anatomic target of phototherapy for VAs is the paraventricular nucleus (PVN) of the hypothalamus. Two animal studies have shown a significant reduction in post-MI VAs with phototherapy targeted near the cranium to modulate the PVN.^70^ The underlying theory of efficacy in this lies in inhibiting microglial proliferation at the PVN, which occurs upon the onset of VT and is believed to exacerbate the VT storm.^70^ No human studies have tested this anatomic target yet, but a human study of this therapy and target would be of great interest. If it yields positive results, it may be worthwhile to further study its use in combination with another non-invasive neuromodulation therapy targeting the left SG, such as TcVNS or TMS, in patients with incessant VT.

## Conclusion

The role of CANS dysfunction in propagating certain arrhythmias has evolved the CANS into a therapeutic target. The non-invasive neuromodulation techniques reviewed have demonstrated an anti-arrhythmic benefit in animal and human studies, opening a promising future of their integration into the management of AF and VAs. These techniques carry a low-risk profile and do not interfere with invasive or pharmacologic interventions, which would permit their safe integration into established standard care. Larger randomized human trials should aim at determining the magnitude of benefit, biomarkers of treatment efficacy, optimal device settings, and the ideal temporal frequency of therapy.

## Figures and Tables

**Table 1:** Human Studies of Non-invasive Neuromodulation Techniques for the Treatment of Arrhythmias

First Author	Technique	Studied Population	Studied Population Size	Study Design	Key Results from Treatment	Significance
Markman et al.^8^	TMS	VT storm	5	Observational cohort	Significant VT reduction	Large VT burden reduction post-TMS treatment
Markman et al.^9^	TMS	VT storm	26	RCT	Significant VT reduction 72 h post-TMS	Clinically significant improvement, despite not meeting the trial’s primary endpoint
Nonoguchi et al.^10^	SG phototherapy	Healthy volunteers (20), refractory VAs (11)	31	Observational cohort	Reduced serum catecholamines	VT reduction nearly met significance in a small VT population
Stavrakis et al.^28^	TcVNS	Paroxysmal AF	40	RCT	Reduced AF, reduced inflammatory markers	First RCT using TcVNS in humans for arrhythmia treatment
Yu et al.^44^	TcVNS	Following STEMI and PCI	95	RCT	Reduced VT, reduced inflammatory markers	First RCT using TcVNS in humans for VT reduction after ACS
Stavrakis et al.^48^	TcVNS	Paroxysmal AF	53	RCT	Reduced AF, reduced inflammatory markers	First RCT of chronic ambulatory use of TcVNS in humans for arrhythmia treatment
Clancy et al.^49^	TcVNS	Healthy adults	48	Observational cohort	Decreased sympathetic tone	First evidence of sympathetic reduction with TcVNS in humans
Kulkarni et al.^52^	TcVNS	Paroxysmal AF	28	Ancillary study to TREAT AF^48^	TcVNS treatment arm had reduced PWA	Used ECGs from TREAT AF to identify a correlation between PWA and chronic TcVNS use
Sohinki et al.^66^	LL-EMF	Paroxysmal AF	18	RCT	Reduced AF duration, reduced AF-triggering ectopic beats	First RCT using LL-EMF in humans for AF treatment

*Abbreviations:* ACS, acute coronary syndrome; AF, atrial fibrillation; ECG, electrocardiography; LL-EMF, low-level electromagnetic field; PCI, percutaneous coronary intervention; PWA, P-wave alternans; RCT, randomized controlled trial; SG, stellate ganglion; STEMI, ST-segment elevation myocardial infarction; TcVNS, transcutaneous vagal nerve stimulation; TMS, transcutaneous magnetic stimulation; VA, ventricular arrhythmia; VT, ventricular tachycardia.
